# Diagnosis and treatment of colorectal tumors: Differences between Japan and the West and future prospects

**DOI:** 10.1002/deo2.66

**Published:** 2021-11-11

**Authors:** Yutaka Saito, Akiko Ono, Victoria Alejandra Jiménez García, Yasuhiko Mizuguchi, Izumi Hisada, Hiroyuki Takamaru, Masayoshi Yamada, Masau Sekiguchi, Mai Makiguchi, Shigeki Sekine, Seiichiro Abe

**Affiliations:** ^1^ Endoscopy Division National Cancer Center Hospital Tokyo Japan; ^2^ Dept. of Gastroenterology Hospital Clínico Universitario Virgen de la Arrixaca; ^3^ Hospital Universitario Virgen Macarena; ^4^ Molecular Pathology Division National Cancer Center Research Institute

**Keywords:** chromoendoscopy, endoscopic mucosal resection (EMR), endoscopic submucosal dissection (ESD), intramucosal adenocarcinoma, laterally spreading tumors (LSTs)

## Abstract

Dye‐based chromoendoscopy has long been used routinely for endoscopic diagnosis of gastrointestinal tumors including colorectal tumors in Japan. In the West, on the other hand, dye‐based chromoendoscopy was not so commonly used. However, with the development of narrow band imaging (NBI), image‐enhanced endoscopy diagnosis has rapidly increased in the West.

The most critical difference between Japan and the West is the histopathological evaluation of the lesions, which determines a major cause of differences in diagnostic and treatment strategies. In the West, intramucosal adenocarcinoma is not diagnosed until the cancer has invaded submucosal layer. In Japan, on the other hand, cancer is mainly diagnosed based on nuclear and structural atypia, and thus intramucosal adenocarcinoma is diagnosed in lesions that correspond to high‐grade adenoma in the West.

In the West, since intramucosal carcinoma is not diagnosed by pathology, all benign adenomas are treated by piecemeal endoscopic resection, and only cancer invading the superficial submucosal layer is indicated for endoscopic submucosal dissection (ESD). Because of the risk of lymph node metastasis in the deep submucosal invasion, the European Society of Gastrointestinal Endoscopy and American Society for Gastrointestinal Endoscopy guidelines state that only superficial submucosal cancer is an indication for ESD. Unfortunately, it is impossible to selectively extract only superficial submucosal invasive cancer even with the use of magnified NBI and pit pattern observation. Therefore, we think that pathologists need to diagnose intramucosal adenocarcinoma with the potential to invade the submucosal layer based on the nuclear and structural atypia. Consequently, intramucosal adenocarcinoma and superficial submucosal cancers should be considered for en‐bloc ESD.

## INTRODUCTION

Colorectal cancer (CRC) is the third most common cancer occurring in men and the second in women worldwide.^1^ Colonoscopy is considered as the gold standard test to detect and remove colorectal neoplasia and by this, its efficacy to reduce incidence and mortality from CRC has been widely demonstrated.^2^, ^3^

In this short review, we will focus on the diagnosis and treatment of colorectal tumors, highlight the differences between Japan and the West, and discuss the future prospects.

## ENDOSCOPIC DIAGNOSIS

In Japan, dye‐based chromoendoscopy has long been used routinely for endoscopic diagnosis of gastrointestinal tumors including colorectal tumors.^4^

The most commonly used dye is 0.6% indigo‐carmine, which enables to observe the mucosal surface structure, recognize the lesion boundary, and diagnose the pit pattern by using optical magnification.

Although indigo‐carmine spraying is sufficient for differential diagnosis between neoplastic and non‐neoplastic lesions, a detailed diagnosis of the degree of irregularity of type V pit is necessary for diagnosis of cancer depth, and crystal violet (CV) staining is essential for this purpose.^5^

Since CV staining targets early‐stage cancer, it is mainly used in tertiary referral hospitals such as cancer centers and university hospitals in Japan.

Although the carcinogenic effects of CV in rodents have been reported, the results of animal laboratory experiments,^6^ in which a large amount of CV was administered orally over several weeks, were obtained under conditions different from those used in endoscopic diagnosis. Therefore, it is somewhat impossible to determine toxicity risk during endoscopic procedures.

In Japan, CV has been used for endoscopic diagnosis for more than 30 years, and there has not been a single case of carcinogenesis reported. Currently, it is used clinically at tertiary referral hospitals with the patient's consent.

For diagnostic procedures of the gastrointestinal tract, several cc of CV are selectively dropped over the lesion. After diagnosis, the lesion is either endoscopically resected immediately, or the stained area is surgically removed at a later date, thus, the effect of CV is almost negligible.

Recently, methylene blue has also been reported as a safe dye to use for endoscopic diagnosis.^7^ Methylene blue is also used in submucosal injection materials with CE‐marking approved;^8^ therefore, CV would be equally safe, as it is a substance which enables nuclear staining similarly to methylene blue.

In the West on the other hand, dye‐based chromoendoscopy was not so commonly used in the past. However, with the development of narrow band imaging (NBI),^9^ image‐enhanced endoscopy (IEE) diagnosis has rapidly increased in the West, since it is now possible to make a diagnosis similar to that of pit pattern diagnosis with a single touch operation.

Still, optical magnification endoscopes are not widely available in the West, and NICE classification,^10^ which can be used without optical magnification, is generally used for NBI classification. Furthermore lesions surface observation for diagnosis of cancer depth is not so common in the West, instead, endoscopic ultrasonography (EUS) is widely used for this purpose .

One of the most critical differences between Japan and the West is the histopathological evaluation of the lesions, which determines a major cause of differences in diagnostic and treatment strategies. The biggest difference is that in the West, intramucosal adenocarcinoma is not diagnosed until the cancer has invaded the submucosal layer. In Japan, on the other hand, cancer is mainly diagnosed based on nuclear and structural atypia, and thus intramucosal adenocarcinoma is diagnosed in lesions which correspond to high‐grade adenoma in the West.

We believe that this cancer definition's difference leads to the major difference in the endoscopic treatment policy.^11^

## ENDOSCOPIC TREATMENT

There are major differences in endoscopic treatment strategies for early‐stage cancer between Japan and the West due to differences in pathological diagnostic criteria as described before.

In Japan, intramucosal carcinoma is considered as cancer, and from the viewpoint of its potential to invade the submucosal layer, en bloc resection by endoscopic submucosal dissection (ESD) is often chosen.^11^ ^–^ ^12^

On the other hand, in the West, intramucosal carcinoma is considered as advanced adenoma, and piecemeal resection of laterally spreading tumors (LSTs) even for a large tumor is accepted or recommended.^11^, ^13^

## ESD FOR LST‐GRANULAR TYPE

In Japan, LST‐granular type (LST‐G) was previously treated by scheduled piecemeal endoscopic mucosal resection (p‐EMR; resecting the area including the large nodule first, and then, piecemeal resection for the remaining flat area) because the submucosal invasion rate was reported lower than that of LST‐non granular type (NG), and the submucosal invasion area could be predicted by endoscopic findings such as large nodules and/or depressions.^14^ However, long‐term surveillance data of these p‐EMR cases showed a recurrence of 1.3% (2/154) invasive cancer after p‐EMR.^15^ Consequently, we chose to perform en bloc resection by ESD even for LST‐G when the tumor size is larger than 3 cm in diameter considering the submucosal invasion rate.

**FIGURE 1:**
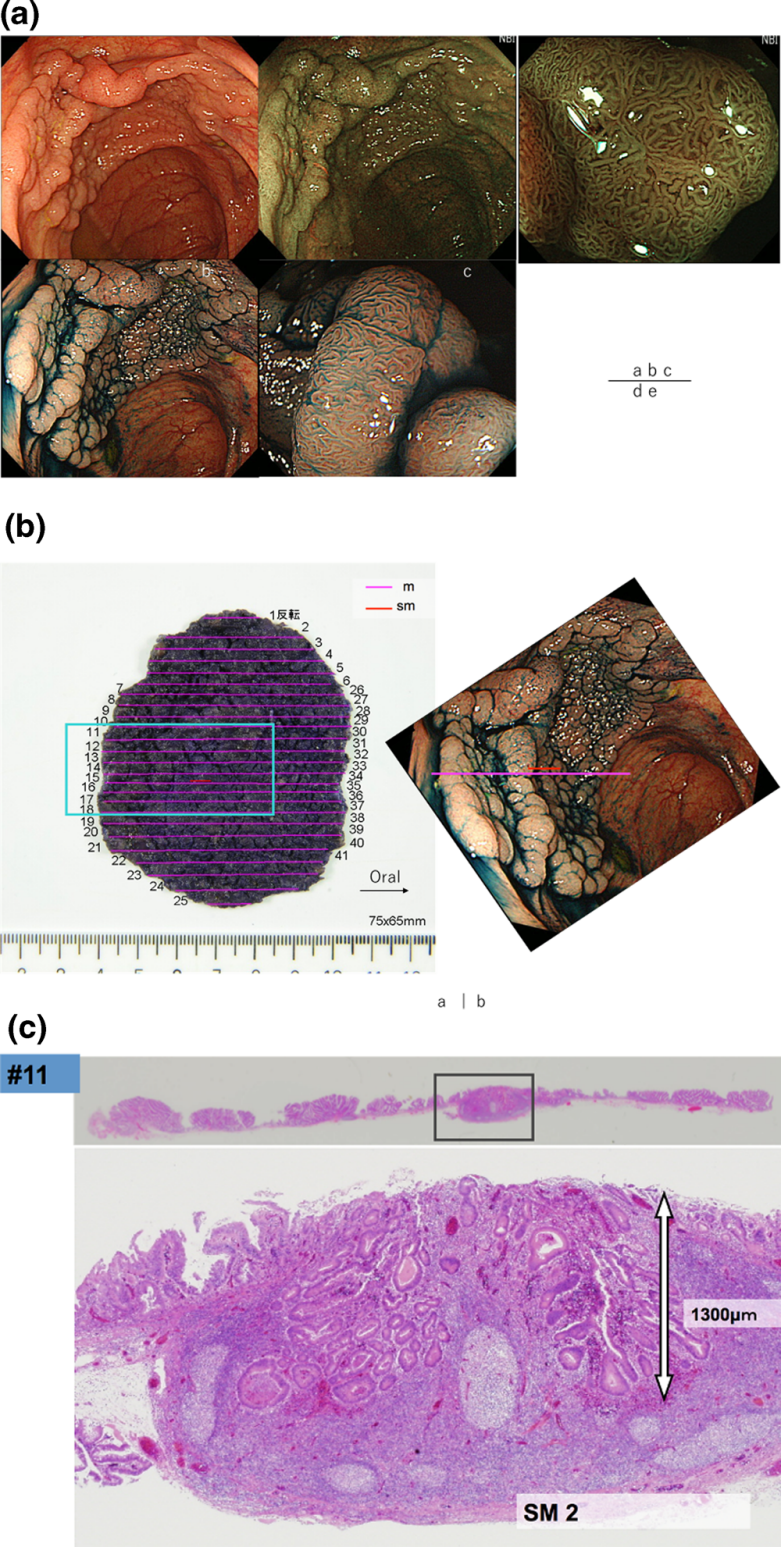
(A) Endoscopic images of A laterally spreading tumor‐granular type (LST‐G) nodular mixed type located in the cecum. (a) White light image; An LST‐G nodular mixed type located in the cecum. (b) Narrow band imaging (NBI) revealed the tumor margin clearly. (c) Magnified NBI revealed a regular vessel and surface pattern and Japan NBI Expert Team (JNET) type 2A was diagnosed. (d) Indigo‐carmine dye was sprayed, and the tumor surface structure was clearly observed. (e) A magnified observation on the large nodule showed type IV pit pattern, and there was no endoscopic finding for submucosal invasion. (B) An en‐bloc resection was achieved due to the large tumor size of 75 × 65 mm. (a) The resected specimen was pined out and cut into 41 sections. Submucosal invasive cancer was diagnosed in the two red lines area. (b) Comparison between resected specimen and endoscopic pictures. Retrospectively reviewed, these submucosal invasion areas were difficult to predict before the treatment. (C) In section 11, the submucosal invasion was 1300μm from the tumor surface due to the destruction of muscularis mucosae

**FIGURE 2:**
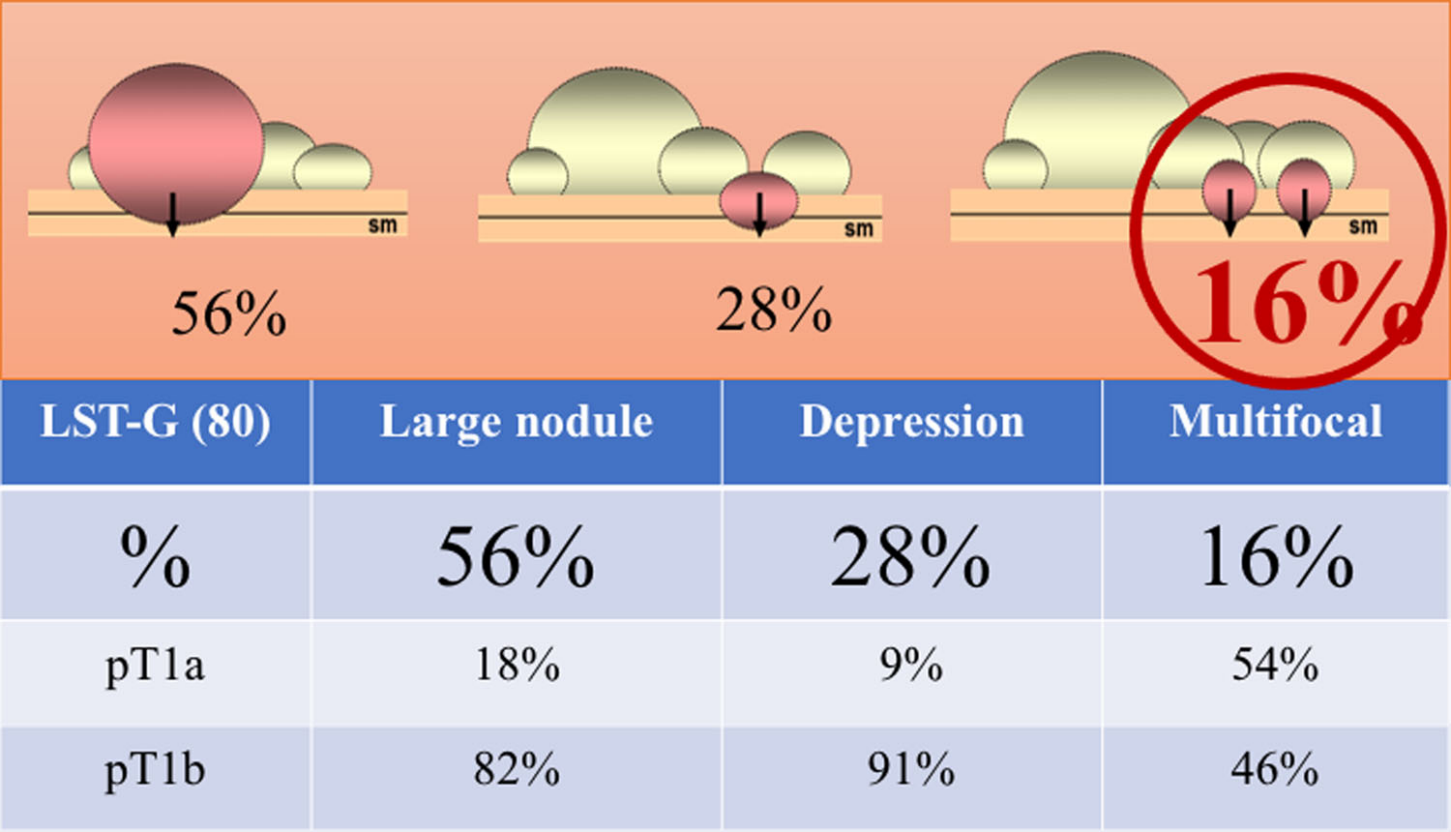
Submucosal invasion rate and invasion pattern in laterally spreading tumor‐granular type (LST‐G). Sixteen % submucosal invasions were diagnosed multifocally outside the area of large nodule or depressed component even in LST‐G, and it was difficult to predict the submucosal invasion area before endoscopic resection even using JNET and maginifed pit pattern observation

**FIGURE 3:**
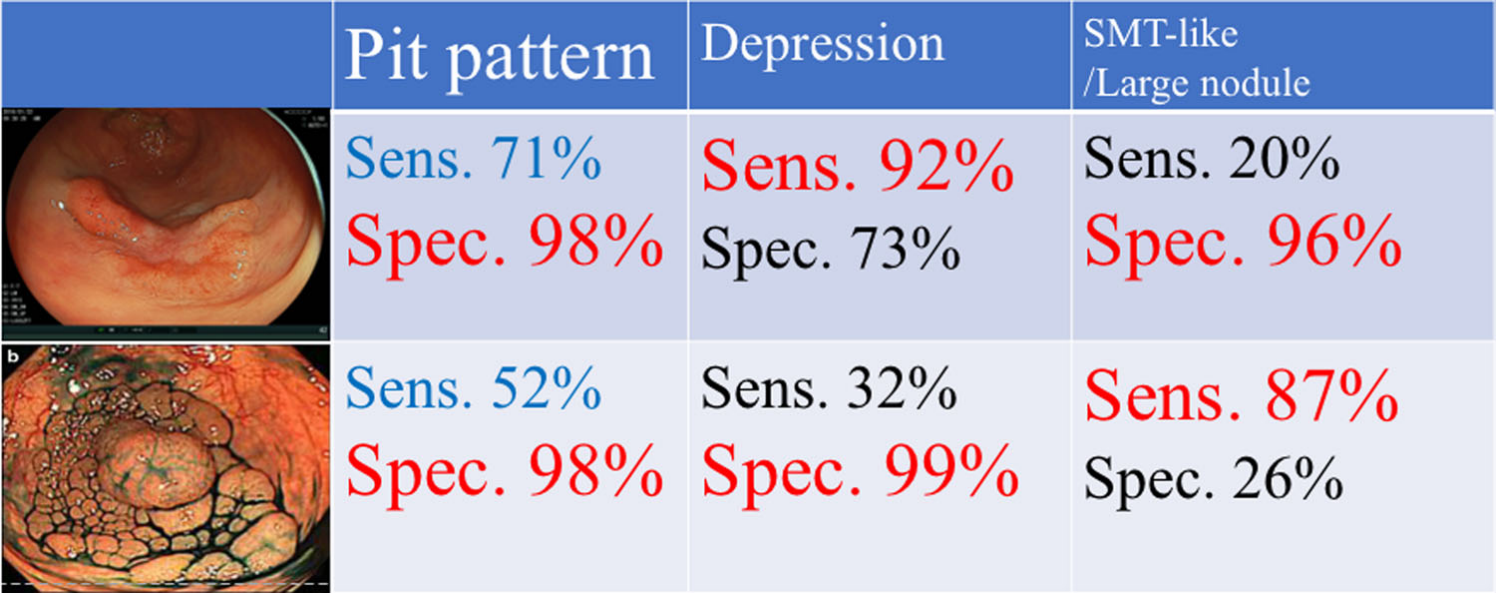
Pit pattern observation shows a higher diagnostic accuracy compared to the other endoscopic findings; however, it is important to understand the limitation of pit pattern observation specially for laterally spreading tumor‐granular type (LST‐G). The sensitivity of pit pattern observation to diagnose submucosal invasion was just 52% for LST‐G, and this means that half of submucosal invasive LST‐G shows non‐invasive pit pattern by magnified diagnosis

**FIGURE 4:**
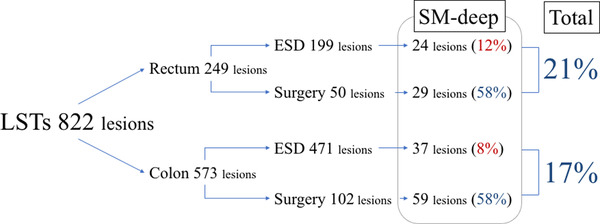
Deep submucosal invasion rate in laterally spreading tumors (including adenoma, intramucosal, and submucosal invasive cancers) treated by surgery and endoscopic mucosal resection /endoscopic submucosal dissection. There was no submucosal invasion rate difference between rectum and colon (21% vs. 17%)

As we began to perform en bloc resections for LST‐G (Figure 1), it became clear that there were some cases of submucosal invasions that could not be diagnosed preoperatively (Figure 1), and presently submucosal invasion rate is estimated higher than before (19%^14^ vs. 7%^16^) (Figures 2, 3, and 4).

In other words, there is a possibility that submucosal invasion could not be determined by histology when the resection was performed in multiple pieces.

It has been later reported from the West that when LST is resected in piecemeal, recurrence as invasive cancer occurs in 4.3% (6/138), thus, concluding that en bloc resection is preferable considering such results.^17^

## SELECTION OF ESD BY TUMOR LOCATION‐COLON VERSUS RECTUM

Regarding the submucosal cancer rate by tumor location, the Australian Colonic Endoscopic (ACE) resection study group reported that the rectum is an indication for ESD because of its high submucosal cancer rate, and the proximal colon has a low submucosal cancer rate.^18^

However, our analysis, including surgical cases, showed no difference in the percentage of submucosal cancer between the colon and rectum (Figure 4).^14^

The ACE group's results included only EMR cases and not surgical cases, which may indicate the possibility of selection bias. In the rectum, EMR is performed aggressively even when submucosal invasion is suspected, whereas in the proximal colon, surgery is often chosen when submucosal invasion is suspected.

## INDICATIONS FOR ESD

In the West, since intramucosal carcinoma is not diagnosed by pathology, all benign adenomas are treated by p‐EMR, and only cancer invading superficial submucosal layer is indicated for ESD.^19^

Because of the risk of lymph node metastasis in deep submucosal invasion,^20^, ^21^ the European Society of Gastrointestinal Endoscopy and American Society for Gastrointestinal Endoscopy guidelines state that only superficial submucosal cancer is an indication for ESD.^19^, ^22^

Is it possible to selectively extract only superficial submucosal invasive cancer? Unfortunately, the answer to this question is no, even with the use of magnified NBI^9^ and pit pattern observation^14^ (Figures 1, 2, and 3), and the answer is probably the same even with EUS.

Therefore, we think that pathologists need to diagnose intramucosal adenocarcinoma (high‐grade adenoma in the West) with the potential to invade submucosal layer based on the nuclear and structural atypia. Consequently, intramucosal adenocarcinoma and superficial submucosal cancers should be considered for en‐bloc EMR/ESD.

This may be controversial in the West, but it is our conclusion based on more than 20 years of experience in pit pattern diagnosis.

## FUTURE PROSPECT

In a near future, dye‐based chromoendoscopy and magnifying endoscopy will need to be routinely used in the West. With the development of artificial intelligence (AI),^23^ the time will soon come when expert diagnosis can be performed easily and appropriately by non‐expert and Western endoscopists.

In addition, it is necessary to reduce the number of unfortunate outcomes in patients, such as recurrence of invasive cancer, by reducing the increasing number of p‐EMRs for large LSTs.

ESD can now be performed safely and easily due to the development of ESD devices and an established ESD strategy. It is necessary to further promote the use of en bloc resection, including ESD, worldwide.

## CONFLICT OF INTEREST

Seiichiro Abe is an associate editor of DEN Open.

## FUNDING INFORMATION

The National Cancer Center Research and Development Fund, Grant Numbers: 29‐A‐13 and 2020‐A‐12.
